# Identification of an immune-related gene prognostic index for predicting prognosis, immunotherapeutic efficacy, and candidate drugs in amyotrophic lateral sclerosis

**DOI:** 10.3389/fncel.2022.993424

**Published:** 2022-12-15

**Authors:** Caihui Wei, Yu Zhu, Shu Li, Wenzhi Chen, Cheng Li, Shishi Jiang, Renshi Xu

**Affiliations:** ^1^Department of Neurology, Jiangxi Provincial People’s Hospital, Medical College of Nanchang University, Nanchang, Jiangxi, China; ^2^Department of Neurology, The First Affiliated Hospital of Nanchang University, Nanchang, Jiangxi, China; ^3^Department of Neurology, The First Affiliated Hospital of Nanchang Medical College, Nanchang, Jiangxi, China

**Keywords:** amyotrophic lateral sclerosis, prognosis, peripheral immune cells, immune signatures, immunomodulatory response

## Abstract

**Rationale and objectives:**

Considering the great insufficiency in the survival prediction and therapy of amyotrophic lateral sclerosis (ALS), it is fundamental to determine an accurate survival prediction for both the clinical practices and the design of treatment trials. Therefore, there is a need for more accurate biomarkers that can be used to identify the subtype of ALS which carries a high risk of progression to guide further treatment.

**Methods:**

The transcriptome profiles and clinical parameters of a total of 561 ALS patients in this study were analyzed retrospectively by analysis of four public microarray datasets. Based on the results from a series of analyses using bioinformatics and machine learning, immune signatures are able to be used to predict overall survival (OS) and immunotherapeutic response in ALS patients. Apart from other comprehensive analyses, the decision tree and the nomogram, based on the immune signatures, were applied to guide individual risk stratification. In addition, molecular docking methodology was employed to screen potential small molecular to which the immune signatures might response.

**Results:**

Immune was determined as a major risk factor contributing to OS among various biomarkers of ALS patients. As compared with traditional clinical features, the immune-related gene prognostic index (IRGPI) had a significantly higher capacity for survival prediction. The determination of risk stratification and assessment was optimized by integrating the decision tree and the nomogram. Moreover, the IRGPI may be used to guide preventative immunotherapy for patients at high risks for mortality. The administration of 2MIU IL2 injection in the short-term was likely to be beneficial for the prolongment of survival time, whose dosage should be reduced to 1MIU if the long-term therapy was required. Besides, a useful clinical application for the IRGPI was to screen potential compounds by the structure-based molecular docking methodology.

**Conclusion:**

Ultimately, the immune-derived signatures in ALS patients were favorable biomarkers for the prediction of survival probabilities and immunotherapeutic responses, and the promotion of drug development.

## Introduction

As a devastating neurodegenerative disease, amyotrophic lateral sclerosis (ALS) results in rapid degeneration of motor neurons and tends to kill victims mainly through ventilatory failure (Munsat et al., 1988; DiPALS Writing Committee, and DiPALS Study Group Collaborators, 2015), for which no effective therapy exists to improve the quality of life, or avoid or reverse the disease progression (Ravits and La Spada, 2009; Gouel et al., 2022). A substantial amount of phenotypic variability has been documented in ALS (Hoyer, 2001; Groeneveld et al., 2003; Gijselinck et al., 2016), including the onset sites, ages of symptom onset and rates of disease progression, which poses a major challenge for clinical practice and clinical trials (Magen et al., 2021). Therefore, there is a strong demand for finding accurate predictors of disease progression to identify different subtypes, and to conduct personalized therapy by distinguishing the subtypes. Ideal biomarkers should exhibit high specificity and sensitivity to discriminate survival benefit during the progress and prognosis of disease course. To date, numerous studies have identified a few biomarkers to distinguish ALS from healthy individuals, frontal temporal dementia, and other neurodegenerative diseases (Thompson et al., 2018; Calvo et al., 2019; Yamada et al., 2021); furthermore, biomarkers developed by some of these studies, are of complexity and difficulty in access of tissue, and measurement (Agosta et al., 2018; Thompson et al., 2018; Agnello et al., 2021; Song et al., 2021). As a result of these studies, and given the fact that ALS is increasingly being viewed as a multisystem disease, the development of a panel of biomarkers that can reflect accurately the characteristics of pathology is seen as a critical goal, not only for diagnostic purposes but also for prognostic and predictive purposes. Currently, few previous researches have explored survival biomarkers of response to therapy. Therefore, further markers are needed to improve survival related prediction and therapy for the realization of more effective clinical practices and trials.

Recently, immunological and inflammatory responses have attracted a great deal of attention, even though the evidence relating to the role that these processes both in ALS pathogenesis and its treatment is highly conflicting. More and more immune targets are being discovered, and immune-modulating therapy has been a potential therapeutic strategy for ALS and neurodegenerative diseases (Angelini et al., 2020; Murdock et al., 2021). In order to individualize immune inflammatory modulation, it would be advantageous to identify potential prognostic biomarkers that are associated with treatment benefits.

**FIGURE 1:**
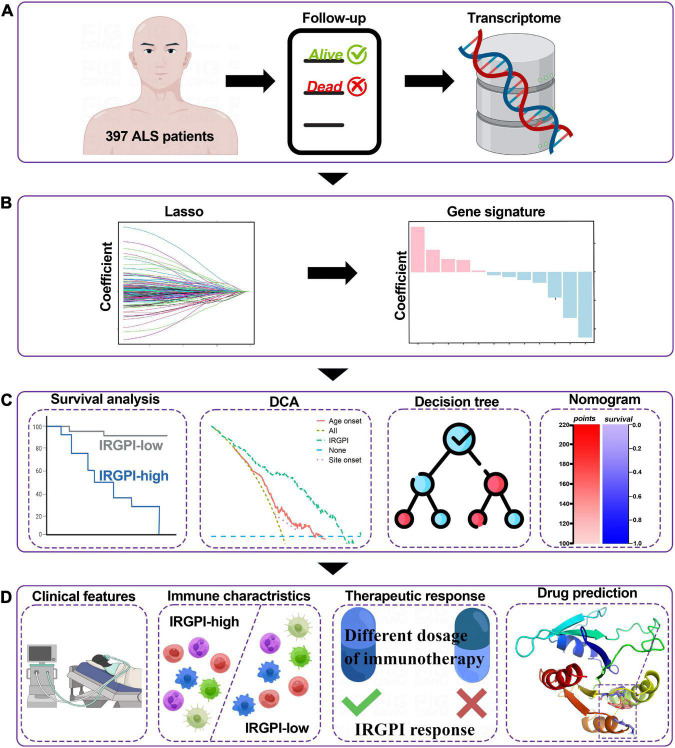
Graphical abstract for the comprehensive study design, and the drawing was assisted by Figdraw (www.figdraw.com). **(A)** As a retrospective analysis of 397 patients with amyotrophic lateral sclerosis (ALS), this study evaluated the transcriptomic profiles and the clinical parameters. **(B)** Least absolute shrinkage and selection operator (LASSO) Cox algorithm was employed to construct immune gene signatures for prognosis. **(C)** Different cohorts and methods were used to validate the prognostic and predictive capabilities. **(D)** Comprehensive analyses of enriched pathways, immune landscape, therapeutic responses, and drug prediction.

It was the aim of this study to develop a prognostic model for ALS that would contemplate the prognosis of immunotherapy. By analyzing the transcriptome data of ALS patients derived from gene expression omnibus (GEO) public database in training cohorts, we identified all immune-related genes (IRGs) in the transcriptome and screened immune-related hub genes associated with patient prognosis for the construction of an immune-related gene prognostic index (IRGPI) in training cohort patients. Significant prognostic values of this index were further confirmed in the testing cohort patients. The IRGPI was also characterized for its intrinsic molecular subtypes, and its immunologic profiles, as well as its ability to prognosticate prognosis in immunotherapy. The schematic flow chart of the meta-analysis is illustrated in Figure 1. Results from the present study showed that the IRGPI was a promising biomarker for immunotherapy which would help to determine their prognostic outcomes.

## Materials and methods

### Data processing and normalization

A series of systematic computerized searches of GEO database^1^ as well as ArrayExpress database^2^ was conducted in order to identify gene expression profiles and clinical information regarding ALS. Two ALS cohort (GSE112676 and GSE112680) (van Rheenen et al., 2018; Swindell et al., 2019) consisting of 397 ALS patients with accessible outcomes and overall survival (OS) times, and one public ALS cohorts (GSE163560) (Giovannelli et al., 2021) of immunotherapy in 107 ALS patients were transcriptomic microarray datasets which was downloaded from the GEO database. To avoid platform-specific effects and confounding batch, the processing and normalization methods of GSE112676 and GSE112680 were described by previous study (van Rheenen et al., 2018; Swindell et al., 2019). Briefly, background correction and the followed quantile normalization, were both performed by conducting the normal–exponential convolution model using neqc function in the R package limma (version 3.48.3). The probes were annotated with gene symbols using the R package illuminaHumanv3.db (version 1.26.0). The apparent batch effect was removed with the ComBat algorithm in the R package sva (version 3.40.0), and then were verified in the visualizations of principal components analysis (PCA) plots. In the GEO cohort (GSE163560), the normalization using the signal space transformation robust multi-chip analysis method, quality control, and log2 transformation was processed according to previous study (Giovannelli et al., 2021), and one sample failing RNA quality control was therefore excluded from the further analysis. To explore more clinical features of ALS, the present study also employed an ALS cohort of transcriptomic microarray dataset comprising 57 ALS patients in the ArrayExpress database (E-TABM-940) (Lincecum et al., 2010), and then subjected to log2 normalization. The dataset probe was annotated by R package hgu133plus2.db (version 3.13.0).

### Development and verification of the IRGPI

A total of 397 ALS patients with survival times and survival outcomes were randomized into training (*n* = 278) and testing cohorts (*n* = 119). IRGs has been collected from the ImmPort database^3^ (Bhattacharya et al., 2018) and the InnateDB database^4^ (Breuer et al., 2013). In the training cohort, all IRGs were applied to Kaplan–Meier (K-M) survival analysis (R package survival, version 3.2-13) to obtain immune-related prognostic genes (IRPGs), and then IRPGs were subjected to develop an IRGPI by applying the least absolute shrinkage and selection operator (LASSO) Cox model *via* the R package glmnet (version 4.1-4). For tuning parameter selection and preventing overfitting in the LASSO model, 10-fold cross-validation, λ = 0.1817, α = 0.2, and γ = 0.999 was chosen in the training cohort. The IRGPI of each sample was calculated by multiplying the expression values of certain genes by their coefficient in the LASSO model and then adding them together: [$I\,R\,G\,P\,I=\sum_{i=1}^{n}C\,o\,e\,f\,\left(m\,R\,N\,A_{i}\times E\,x\,p\,\left(m\,R\,N\,A_{i}\right)\right)$]. Then, the patients were divided into an IRGPI-high subtype (above the median) and IRGPI-low subtype (below the median) by the median risk score. In the training and testing cohorts, time-dependent receiver operating characteristic curve (ROC) curves were drawn to evaluate the IRGPI in the prediction of prognostic accuracy with R package time ROC (version 0.4). For the purpose of validating the independent prognosis value of the IRGPI and clinical features, multivariate Cox proportional hazards regression analysis was performed with R package survival. K-M survival curves were generated between the subtypes of site-onset, age-onset and the IRGPI in the training cohort, and compared with a log-rank test.

### Construction and validation of ALS prognostic nomogram and decision tree

We developed a prognostic nomogram predicting OS based on the Cox proportional hazard regression model by using the R package rms (version 6.2-0) and survival. The nomogram was constructed to estimate 1-, 3-, 5-, 7-, and 10-year survival probabilities. In the training and testing cohorts, the accurateness of the nomogram was estimated by the concordance index (C-index) *via* a 1,000-repeat bootstrap validation method, and was evaluated graphically by calibration chart between predicted and actual survival outcome, the decision curve analysis (DCA) comparing clinical utility among the nomogram at median survival time, and time-dependent area under the curve (tAUC) in the training and testing cohorts at time points of 2, 3, 5, 7, and 8 years. Apart from the R package survival, the C-index, calibration chart, DCA, and tAUC was respectively conducted by using the R package of pec (version 20220306), rms, ggDCA (version 1.2), and riskRegression (version 2021.10.10). Risk stratification was developed using a decision tree *via* recursive partitioning analysis (RPA) based on the cumulative point score of the ALS nomogram with R package rpart (version 4.1.16).

### Comprehensive analyses of the immunological and molecular characteristics in the IRGPI subtypes

To analyze whether there were differences in the immune cell abundance of blood samples between IRGPI-high and IRGPI-low patients, the evaluation of the relative infiltrating abundance of 22 immune cells from gene expression profiles of ALS patients in the training cohort was conducted using CIBERSORT algorithm with 1,000 permutations (Newman et al., 2019).

### Evaluation of the IRGPI to the therapeutic response

The responses of the IRGPI to treatments in ALS patients who received immunotherapy was examined in the GEO cohort (GSE163560). The cohort included the messenger RNA (mRNA) expression levels of blood samples from patients accepting placebo-controlled trial of IL2 immunotherapy injection at different days and with two dose levels: 1MIU and 2MIU. The results were confirmed by the visualization of barplots and waterfall plot.

### Molecular docking

To explore more immune therapeutic drugs, docking analysis was applied to identify the potent *de novo* drug for ALS. Firstly, protein-protein interaction (PPI) networks were constructed based on the identified differentially expressed genes (DEGs) by using the STRING database^5^ with a combined score > 0.7 (Szklarczyk et al., 2017). Secondly, Cytoscape software (version 3.9.1) (Shannon et al., 2003) was used to identify and visualize the hub genes in the PPI networks within immune genes; the hub gene was selected as the core target with MCODE plugin in Cytoscape. Thirdly, the ligand-binding pocket of the core target was predicted with the ProteinsPlus^6^, which was a web-based molecular modeling tool. Finally, molecular structures of 11,797 small molecules and the core target, were respectively collected from DrugBank^7^ (Wishart et al., 2018) and PDB database^8^ (Berman et al., 2000). The binding mode and affinity between small molecular ligands and the core target were obtained using the Autodock Vina (Trott and Olson, 2010). The capabilities of small molecules to penetrate blood-brain barrier (BBB) were validated by DrugBank.

### Statistical analyses

Duration of OS was defined as the period from disease onset to death, tracheostomy or non-invasive ventilation more than 23 h a day. Survival analyses were censored by the date of the last check. All statistical analyses and visualizations were performed by using the R software (version 4.1.0). All data variables were described as means ± SD (continuous variables), or as frequencies and percentages (categorical variables). Continuous variables were compared using non-parametric Wilcoxon tests with an irregular distribution or Student’s *t*-tests with a normal distribution. The K-M plot was performed to present survival curves for clinical features, and the log rank test were used to evaluate statistically differences. The correlation analyses were based on Pearson correlation. A Pearson correlation *R* > 0.7 indicates a strong linear correlation, a correlation 0.5 < *R* ≤ 0.7 indicates a substantial linear correlation, a correlation 0.3 < *R* ≤ 0.5 indicates a weak linear correlation, and a correlation *R* ≤ 0.3 indicates no linear correlation. *P* < 0.05 was considered to be statistically significant.

## Results

### Construction and validation of the IRGPI in the ALS cohort

**FIGURE 2:**
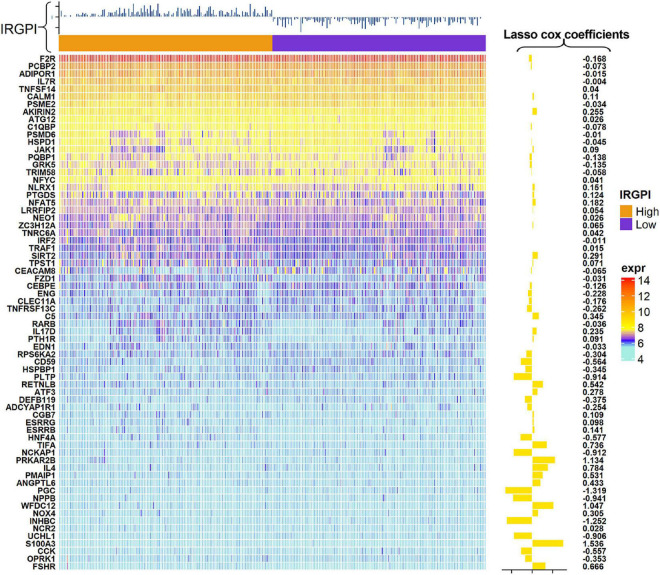
Heatmap showed the landscape of expression levels of the immune-related gene prognostic index (IRGPI) signature and the LASSO COX coefficients.

**FIGURE 3:**
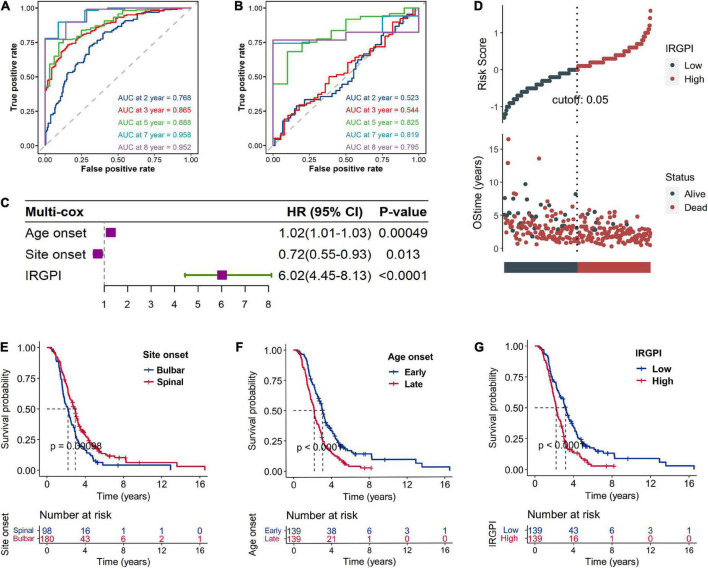
Validation of the immune-related gene prognostic index (IRGPI) signature based on the testing cohort. The time-dependent ROC curves exhibited that the IRGPI signatures were with convincible performances in the training cohort **(A)** as well as the testing cohort **(B)**. Independent prognostic value of the IRGPI and clinical features was evaluated by multivariate Cox proportional hazard regression which was intuitively displayed in the forest plots **(C)**. The risk plot **(D)** demonstrated a clear distinguishment of survival status between IRGPI-high and -low subtypes with red dots representing dead patients and black ones alive, and shown the survival status becoming worse as the risk value increased. Kaplan–Meier (K-M) survival curves demonstrated that patients in the group of bulbar site-onset **(E)**, late age-onset **(F),** and IRGPI-high **(G)** had significantly worse overall survival (OS) than their counterpart. Number at risk is number of subjects exposed to endpoint risk at various time points, and the number of subjects exposed to the risk of endpoint events began to decrease over time.

A total of 2,720 IRGs has been collected from ImmPort database^9^ (Bhattacharya et al., 2018) and the InnateDB database^10^ (Breuer et al., 2013). mRNA-seq analyses were conducted with sample of whole blood of ALS patients. PCA showed the procession of removing batch effect (Supplementary Figures 1A, B). In the ALS training dataset, sixty-eight genes, as the IRGPI signatures, were screened by the LASSO COX algorithm with their individual non-zero coefficients which were shown in the heatmap (Figure 2). Moreover, the heatmap also showed the landscape of different expression levels of the IRGPI signatures between the IRGPI-high and -low subtypes. LASSO plot (Supplementary Figure 2A) and Lambda plot (Supplementary Figure 2B) were carried out to prevent overfitting. The time-dependent ROC curves exhibited that the IRGPI signatures were with convincible performances in the training cohort as well as the testing cohort (Figures 3A, B), achieved AUC of 0.768, 0.865, 0.888, 0.958, and 0.952 at 2, 3, 5, 7, and 8 years in the training cohort, and achieved AUC of 0.523, 0.544, 0.825, 0.819, and 0.795 at 2, 3, 5, 7, and 8 years in the testing cohort. Independent prognostic value of the IRGPI and clinical features was evaluated by univariate Cox and multivariate Cox proportional hazard regression, and was intuitively displayed in the forest plots (Figure 3C). The forest plot of the multivariate Cox analysis showed that the IRGPI was the most striking risk factor among clinical features (hazard ratio = 6.02, 95% CI = 4.45–8.13, *P* < 0.0001). The risk plot (Figure 3D) demonstrated a clear distinguishment of survival status between IRGPI-high and -low subtypes with red dots representing dead patients and black ones alive, and showed the observed OS becoming worse as the risk value increased. The purpose of risk plot is to help make smarter decisions by comparing different risks against each other. IRGPI was a continuous variable that was divided into IRGPI-high (above the IRGPI median) and IRGPI-low (below the IRGPI median) groups by the median. Similarly, age-onset was a continuous variable that was divided into early age-onset (below the age-onset median) and late age-onset (above the age-onset median) groups by the median. K-M survival curves demonstrated that patients in the group of bulbar site-onset (Figure 3E), late age-onset (Figure 3F), and IRGPI-high (Figure 3G) had significantly worse survival than their counterpart (*P* < 0.0001).

### The performance of the nomogram and the decision tree in risk stratification

**FIGURE 4:**
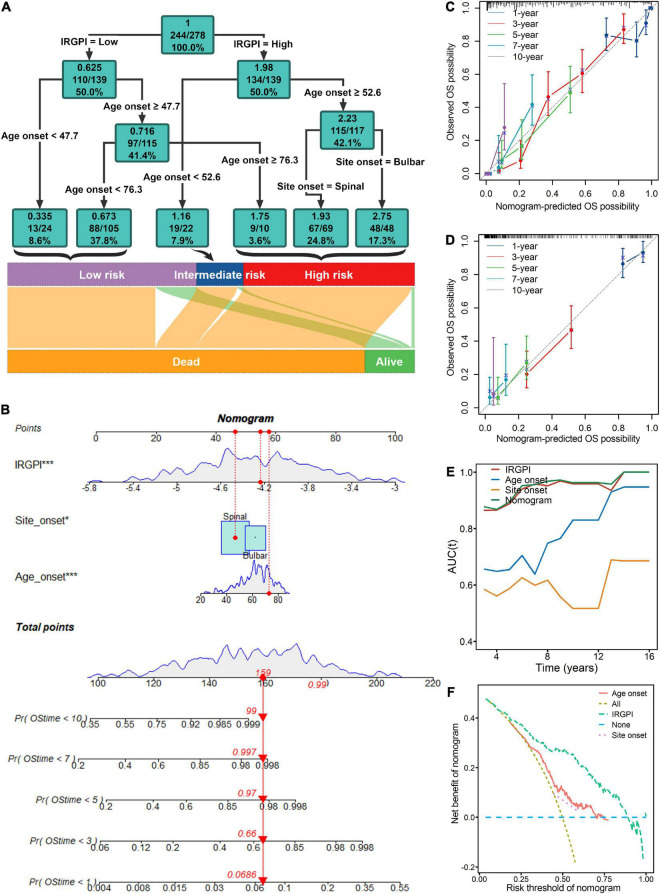
Risk stratification and survival prediction are improved by combining immune signatures of the immune-related gene prognostic index (IRGPI) with clinical features. **(A)** The decision tree was developed to enhance risk stratification. **(B)** The nomogram was created for the quantitative risk evaluation of individual patients. Calibration curves for 1-, 3-, 5-, 7-, and 10-years showed effective consistency between the prediction and observation in the survival probability in the training **(C)** and testing cohort **(D)**. **(E)** Both the nomogram and the IRGPI in time-dependent area under the curve (tAUC) plot were higher than the clinical features of age onset and site onset, which suggested that the nomogram and the IRGPI led a greater prediction performance. **(F)** Decision curve analysis (DCA) demonstrated that the nomogram had improved net benefits at a range of clinically reasonable risk thresholds, which had effective clinical application prospects for making valuable and informed judgments of the prognosis. *P < 0.05; ***P < 0.001.

The multivariate Cox analysis indicated that IRGPI, site onset and age onset were independent prognosticator of mortality in ALS patients (Figure 3C). Thus, the nomogram (Figure 4B) incorporating these factors, was developed to investigate predicted probabilities of patient survival with acceptable accuracy at time points of 1, 3, 5, 7, and 10 years. The evaluated accuracy of nomogram performance in discriminating risk stratification, based on calibration curves, tAUC, and C-index. In the training and testing cohort, nomogram to predict OS probabilities at time points 1, 3, 5, 7, and 10 years was evaluated by calibration curves which showed effective consistency between the observed OS probabilities and predict OS probabilities (Figures 4C, D). Respectively, the C-index for the training and testing cohort was 0.747 (*P* < 0.001) and 0.642 (*P* < 0.001). Both the tAUC of nomogram or the IRGPI were higher than the clinical features of age onset and site onset, which suggested that the nomogram and the IRGPI led a greater prediction performance (Figure 4E). DCA (Figure 4F) demonstrated that the nomogram had improved net benefits at a range of clinically reasonable risk thresholds, which had effective clinical application prospects for making valuable and informed judgments of the prognosis. The nomogram model’s clinical applicability or simplicity of use in a wide range of healthcare systems is its most appealing feature. As an example (Figure 4B), an individual, no matter male or female, onset at age 73 years old whose lesions was in the spinal and the IRGPI score was −4.5, would have a total risk score of 159 points, corresponding to a 1-, 3-, 5-, 7-, and 10-year survival probability of 6.9, 66, 97, 99.7, and 99.9% (Figure 4B). Apart from high IRGPI value, later onset ages or bulbar onset site had resulted in shorter-term survival. Finally, classification criteria of the IRGPI, age onset and site onset were to build the decision tree, and three different risk subgroups were well discriminated with quantitative risk assessment for individual ALS patients (Figure 4A). In the decision tree, a green square represented a node. In green squares, the top is the entropy, the middle is the died patients/observed patients, and the bottom is the percentage of observed patients in the node.

### The correlations between the IRGPI and clinical features

**FIGURE 5:**
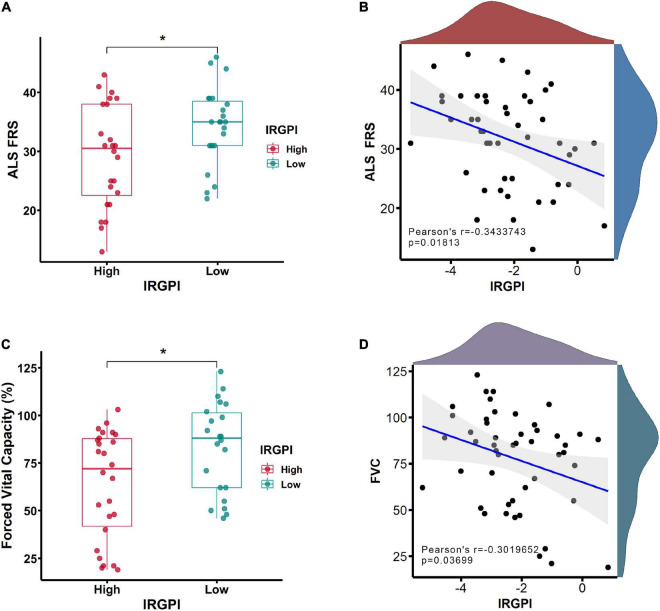
The clinical features of ALS functional rating scale (ALS-FRS) **(A)** and forced vital capacity (FVC) **(C)** were investigated between immune-related gene prognostic index (IRGPI)-high and -low subtypes. The correlation analyses between IRGPI and clinical features of ALS-FRS **(B)** and FVC **(D)**. *P < 0.05.

Clinical features of ALS were investigated between IRGPI-high and -low subtypes within forced vital capacity (FVC) and ALS functional rating scale (ALS-FRS). Compared to IRGPI-high subtype, IRGPI-low subtype had a better performance in ALS-FRS (Figure 5A) and FVC (Figure 5C). The correlation analyses between IRGPI and clinical features was conducted, including the decreasing rate of ALS-FRS (Pearson’s correlation coefficient −0.34; *P* = 0.01813) (Figure 5B) and FVC (Pearson’s correlation coefficient −0.30; *P* = 0.03699) (Figure 5D).

### The immune characteristics in the IRGPI subtypes

**FIGURE 6:**
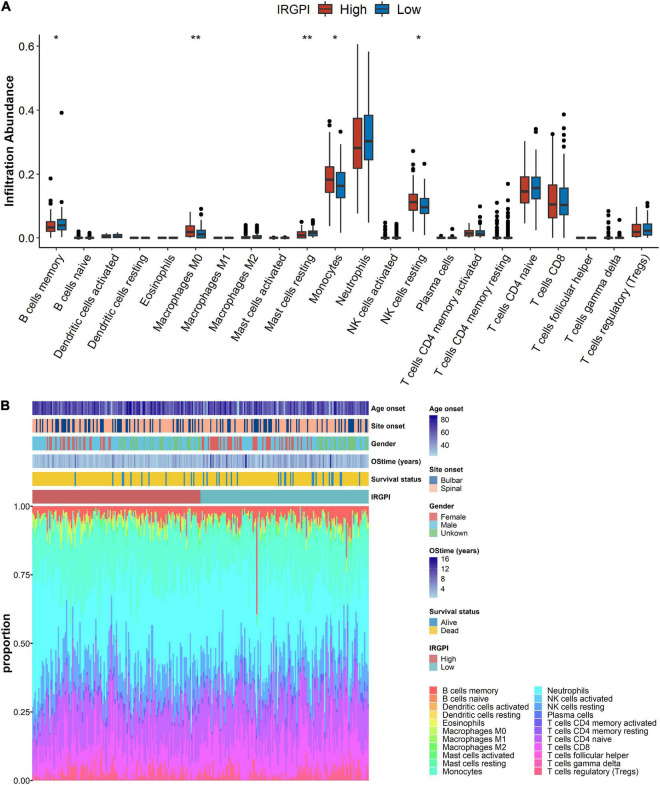
The landscape of the immune microenvironment in ALS and the characteristics of different immune-related gene prognostic index (IRGPI) subtypes. **(A)** The proportions of immune cells between different IRGPI subtypes Wilcoxon test (*p < 0.05, **p < 0.01). **(B)** The IRGPI subtypes and proportions of immune cells for 397 amyotrophic lateral sclerosis (ALS) patients. Age onset, site onset, gender, OStime, and survival status are shown as patient annotations.

Wilcoxon test was utilized to analyze the infiltration abundance of immune cells between IRGPI-high and -low subtypes. We found that Macrophages M0, Monocytes and resting NK cells were more abundant in the IRGPI-high subtype, while memory B cells and resting Mast cells were more abundant in the IRGPI-low subtype (Figure 6A). The immune landscape and its related characteristics of clinical features between IRGPI-high and -low subtypes was shown (Figure 6B).

### The IRGPI response to immunotherapies

**FIGURE 7:**
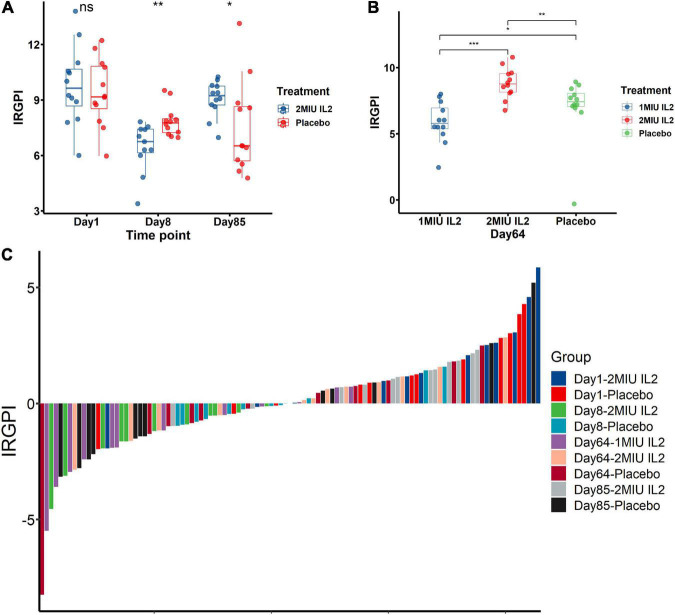
The immune-related gene prognostic index (IRGPI) to therapeutic response in amyotrophic lateral sclerosis (ALS) patients and mice models. The responses and drug sensitivities of the IRGPI to immunotherapy of IL2 injection was shown in the barplots **(A,B)** and the waterfall plot **(C)**. The Wilcoxon test was used to compare the statistical differences all above. *P < 0.05; **P < 0.01; ***P < 0.001.

To investigate potential therapy to improve the IRGPI-related prognosis for all practical purposes, the white blood cells of interleukin-2 (IL-2)-treated and placebo-treated patients were used for transcriptome analyses (Camu et al., 2020; Giovannelli et al., 2021). The potential responses and drug sensitivities of the IRGPI to IL2 immunotherapy was assessed (Figures 7A–C). Interestingly, placebo-treated patients have lower IRGPI at Day 8 and Day 85 than Day 1 (Figure 7A). In the original study, 3 months of riluzole was administrated in placebo-treated patients and IL-2 treated patients before clinical trial entry. Previous studies had confirmed that riluzole reduced immune respose and immune-related biomarkers (Gilgun-Sherki et al., 2003; Liu et al., 2013; Shirani et al., 2016; Rotolo et al., 2021). Therefore, riluzole might be still affected all patients after clinical trial entry, including placebo-treated and IL-2 treated patients, causing lower IRGPI at Day 8 and Day 85 than Day 1. In contrast, the 2MIU IL2 group exhibited a significant decrease in the IRGPI value compared to the placebo group (*P* < 0.01) on the 8th day after IL2 injection (Figure 7A). However, on the 64th and 85th day, 2MIU IL2 group had significantly higher levels of the IRGPI value compared to the placebo group (*P* < 0.05, Figures 7A, B). Interestingly, compared to 2MIU IL2 group, 1MIU IL2 group had a significantly lower level of the IRGPI value on the 64th day (*P* < 0.001) (Figure 7B). The responses of the IRGPI values to different dose of IL2 immunotherapy at different days was also confirmed by the visualization of waterfall plot (Figure 7C). In the present study, the waterfall plot reflected how IL2 treatment affect the IRGPI scores. The bar under the *x*-axis is IRGPI-low scores which were more beneficial for ALS patients than IRGPI-high scores with therapeutic effect. The bar above the *x*-axis is IRGPI-high scores which were harmful for ALS patients. Although the treatment was not effective for all patients, it was effective in some patients. Thus, we suggested short-term therapy (about 8 days) of 2MIU IL2 injection would provide potent clinical benefit for ALS patients, and long-term treatment (about 64 days) of 1MIU IL2 injection may as well be beneficial.

### Core target identification and candidate molecules prediction

**FIGURE 8:**
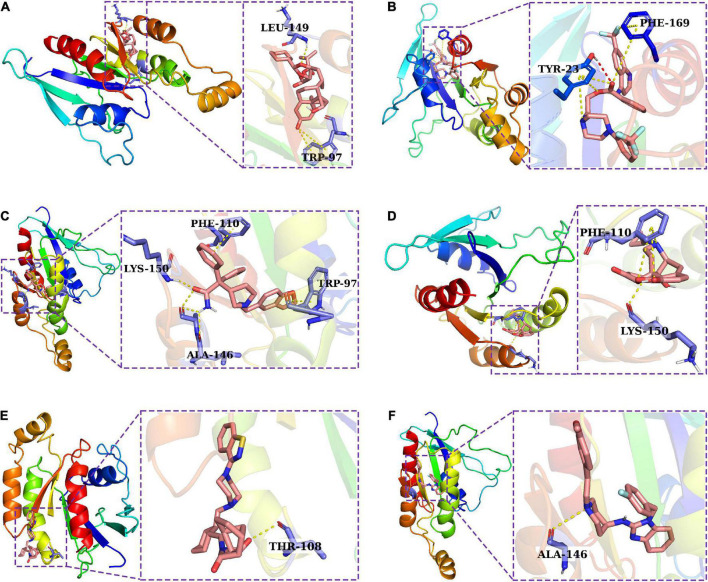
A molecular docking analysis was used to construct docking models for six potential drugs and their intended targets. Three dimensional (3D) structures showing the binding modes between the predicted binding pocket of CDC42 and ciclesonide **(A)**, antrafenine **(B)**, darifenacin **(C)**, naltrexone **(D)**, lurasidone **(E)**, and astemizole **(F)**.

The IRGPI consists of IRGs which were used to develop a PPI network by utilizing the STRING database (Szklarczyk et al., 2017) (confidence score > 0.7). By utilizing MCODE plugin in Cytoscape software, the core target of the IRGPI was determined in the PPI network, and displayed by the Cytoscape (Supplementary Figure 3). The first cluster in MCODE was selected, and CDC42 was located at the core and had the highest degree among them all, making it the core target. Molecular docking is a computer approach for compound screening that is structure-based. In the present study, the structures of 11,797 small molecules were collected from the drugbank database, and subjected to molecular docking. The results showed the top six molecules (Ciclesonide, Antrafenine, Darifenacin, Naltrexone, Lurasidone, and Astemizole) which had the highest affinity with the predicted binding pocket of CDC42. The detailed binding energy was shown in three dimensional (3D) graphics for the six docking models (Figure 8). Darifenacin (DB00496), for example, formed hydrogen bonds with amino acid residues LYS-150 and alanine (ALA)-146. Furthermore, the possible formation of π-π interaction between PHE-110 or TRP-97 residue and the cyclic molecules of the ligand assisted the drug compound in connecting to the active site of CDC42.

## Discussion

Peripheral immunity, is one of the most common pathophysiology of neurodegenerative diseases, which has a communication with neuroimmune system, modulate neuroinflammation, and affect central nervous system (CNS) neurodegeneration (Chitnis and Weiner, 2017; Holzer et al., 2017; Liu Y. et al., 2017; Li et al., 2018). Moreover, peripheral immunity is a pivotal element being increasingly recognized in ALS, and an association has been identified between peripheral immune cell levels and the progression even the rapid progression of ALS (Murdock et al., 2017; Sidaway, 2017; Li et al., 2020). There are both neuroprotective and adverse effects that can be induced by the interactions between microglia and astrocytes in crosstalk with peripheral immune cells (Vahsen et al., 2021). ALS patients have abnormalities in peripheral immune system, which may be due to changes in monocytes, T lymphocytes, cytokines, and complement in their peripheral blood (McCombe et al., 2020). Though therapeutic immunomodulation strategies have proven to be a potential therapy to improve disease outcomes and prolong survival (Yan et al., 2006; Zhao et al., 2020; Zhou et al., 2020; Sun et al., 2021), the overall response rates to immunomodulation therapies are still very low for ALS patients (Beers et al., 2011; Devigili et al., 2011; Komine et al., 2018). Thus, it is crucial to identify ALS patients who may benefit from these therapies with quantifiable indicators of hazard stratification for the improvement of disease outcomes and OS. Currently, little previous research has explored survival biomarkers of response to therapy. Therefore, further markers are needed to improve survival related prediction and therapy for the realization of more effective clinical practices and trials.

Our cohorts seem to be reflective of the larger population of ALS patients with a wide range of survival time and age onset to most of previous studies. Based on the training cohort, the IRGs were applied to K-M survival analysis to obtain IRPGs, and then the IRPGs were subjected to LASSO Cox regression model to construct the IRGPI comprising 64 genetic signatures which are independent prognostic indicators of OS with the validation in the training and testing cohorts. Notably, it was unreasonable that most of the previous studies using LASSO algorithm did not seem to avoid the risk of over-fitting which is a frequent question in machine learning. From the LASSO model, the IRGPI proved to have the effective capacity in discriminating risk stratification with IRGPI-high subtype representing worse survival and IRGPI-low one better, suggesting it is a trustworthy stratification tool in pooled populations and similar-risk groups.

To maximize the usage of the IRGPI, survival decision tree and nomogram, both comprising IRGPI, was constructed. The survival decision tree was constructed for the enhancement of risk stratification on the basis of IRGPI, age onset, and site onset for ALS patients. In the tree, the IRGPI played the major dominant position. Later onset ages tended to have higher risks, and the bulbar onset site also had higher risks of morbidity. The nomogram, a simple-to-use tool, has frequently been used in estimating the likelihood of survival probabilities based on relevant clinical parameters. The nomogram incorporating the IRGPI, age onset, and site onset was developed, and validated in the calibration chart, tAUC, C-index, and DCA, which performed the highest acceptable accuracy in discriminating risk stratification of survival prediction in 1-, 3-, 5-, 7-, and 10-year when compared to other traditional features. Apart from low IRGPI value, later onset ages or bulbar onset site has resulted in shorter-term survival. The nomogram of the current study, relatively straightforward to understand, is anticipated to be a simple and effective tool for clinical practices and may be used to inform patients about their future risk for up to 10 years, based on the results of their IRGPI.

Importantly, the results of immune infiltration analyses were used to understand the immune and molecular characteristics of the IRGPI signature genes. Combined with additional molecular and immunological subtypes, the IRGPI classification might help ALS patients differentiate between molecular and immunological subgroups. The comprehensive understandings of the landscape in the immune microenvironment might contribute to the discovery of novel approaches to treat ALS or to regulate the immune microenvironment for the improvements of the effectiveness in immunotherapies. The infiltrating immune cell abundance profile significantly differed between two IRGPI subtypes. Macrophages M0, monocytes and resting NK cells were more abundant in the IRGPI-high subtype, while memory B cells and resting mast cells were more abundant in the IRGPI-low subtype. Peripheral monocytes and NK cells were involved in ALS pathophysiology (Murdock et al., 2016; Jin et al., 2020; McCombe et al., 2020; Gaur et al., 2021; Figueroa-Romero et al., 2022). NF-κB induces the release of associated inflammatory mediators (Liu T. et al., 2017). In resting mast cells, NF-κB dimers are physically associated with IkB, an inhibitor of NF-κB (Zhou et al., 2016). Hence, the increased proportion of resting mast cells might tend to suppress inflammation. Memory B cells might involve in growth and guidance of neuron which provide potent benefit for ALS (Yu et al., 2008). A cellular therapy of ALS has demonstrated that the reduction of peripheral macrophage activation suppressed proinflammatory microglial responses, delayed symptoms and improved favorable prognosis (Chiot et al., 2020). The high abundance of macrophages M0 might disturb the phenotype balance of M1/M2 macrophage fractions because macrophages M0 might be polarized into either M1 or M2 macrophages. Additionally, memory B cells might extend survival time while Monocytes and resting NK cells might short survival time.

To test the clinical capabilities of the IRGPI in stratification of clinical symptom levels, the ArrayExpress dataset (E-TABM-940) was used, which showed that the IRGPI clearly distinguish different disease levels of ALS. Comparing to IRGPI-high patients, the IRGPI-low patients had better FVC scores of pulmonary function and ranked higher in ALS-FRS.

To date, the biomarker of immunotherapeutic responses remained unmet in clinical practices for ALS patients. To determine whether the IRGPI was a reliable biomarker in prediction of immunotherapeutic responses in ALS patients, the difference was identified in the immunotherapeutic responses between IRGPI-high and -low subtypes.

Interleukin-2 (IL-2) is an immunostimulatory cytokine that is capable of stimulating a wide variety of leukocytes, including T-cells and natural killer cells, so IL-2 is critical in regulating peripheral immunity (Levin et al., 2012). In the GEO cohort (GSE163560), patients receiving IL-2 immunotherapy was described in detail in the previous study (Giovannelli et al., 2021). We found a short-term (about 8 days) therapy of 2MIU IL2 injection might provide some degree of clinical benefit of survival time for ALS patients rather than a long-term (about 64–84 days) treatment. Interestingly, to a certain extent, compared to 2MIU IL2 group, 1MIU IL2 group, a lower dose of IL2 therapy, might be beneficial for survival time of ALS patients in the long-term (about 64 days). To date, it had been proved that low-dose IL-2 (LD-IL-2) enhanced the regulatory T-cell (Treg) in autoimmune conditions (Hotta-Iwamura et al., 2018). However, previous studies did not study the association between LD-IL-2 and survival outcomes of ALS patients although there was a significant increase in Tregs levels after 1 and 2 MIU IL-2 administration (Busse et al., 2010; Asano et al., 2017; Camu et al., 2020; Giovannelli et al., 2021). Based on the prognostic index of the IRGPI, our study gave more details to guide the IL-2 administration on different dosage. To extend survival time, the present study suggested it might be not reasonable for the administration of 2MIU IL2 in the long-term, which should be terminated within the short-term, or reduced the dose of the drug to 1MIU if the long-term therapy was required. The speculated reasons for the poorer performance of the administration of 2MIU IL2 in the long-term might due to the sustained overcorrection of Tregs levels, as is reported that a high percentage of Tregs would lead to a poorer prognosis (Shah et al., 2011; Mao et al., 2012; Tao et al., 2012; Feng et al., 2017). Appropriate dose reduction of LD-IL-2 in the long-term might have a better capability in the balance of Tregs levels.

A useful clinical application for the IRGPI was to screen potential compounds by combining its core target and the structure-based molecular docking methodology. In the PPI network, CDC42 was located at the core and had the highest degree among them all, making it the core target. CDC42 is one of typical the Ras homologous protein (RHO) family members of GTPases whose dysregulation leads to neuronal cell damage (Iguchi et al., 2009; Tönges et al., 2014; Koch et al., 2018). In addition to their critical role in neuronal development and neuronal survival, Rho-GTPases play an important role in cytoskeleton dynamics, and they are therefore vital to axonal regeneration, maintenance, and transport (Kirby et al., 2011; Klemann et al., 2018). Our previous proteomics study in ALS mouse models have revealed that CDC42 has significant differences between pre-onset and age-matched wild type group, and onset and pre-onset group (Zhang et al., 2018), which indicated it might be important in the regulatory mechanisms of ALS. In autoimmune disease, CDC42 can be harnessed as a decisive regulator of peripheral tolerance since it suppresses Th17 aberrant differentiation or pathogenicity, and promotes the differentiation, stability, and function of Tregs, so the CDC42 pathway may play a critical role in the immune processes (Kalim et al., 2018). Thus, it suggested that the disease of ALS is accompanied by abnormal RHO signaling regulated by CDC42 (Jiang et al., 2022). Therefore, the development of effective drugs targeting CDC42 has been intensive while most of drugs targeting CDC42 do not have the ability to penetrate the BBB. In the present study, from 11,797 small molecules, we discovered six drugs that had high affinity for CDC42. These six drugs have excellent performance in gastrointestinal absorption and penetration of the blood-brain barrier. Among them, Astemizole has been reported that immunosuppressive potential of Astemizole against T-cell proliferation and cytokine secretion in macrophages by modulating mitogen-activated protein kinase (MAPK) signaling pathway (Jakhar et al., 2018). As a result of low-dose Naltrexone administration, BV-2 microglia cells acquired a quiescent anti-inflammatory M2 phenotype from highly activated pro-inflammatory phenotypes (Kučić et al., 2021). Furthermore, low-dose naltrexone has been shown to modulate Toll-like receptor four signaling as well as transiently blockend endogenous opioid receptors to reduce glial inflammation (Toljan and Vrooman, 2018). In conclusion, ALS patients had meaningful differences between immune subtypes, with IRGPI-high subtype having a high risk of death. The decision tree and the nomogram, based on the IRGPI, were helpful to guide individual risk stratification. Moreover, The IRGPI may be used to guide preventative immunotherapy for patients at high risks for mortality. The administration of 2MIU IL2 injection in the short-term is likely to be beneficial for the prolongment of survival time, whose dosage should be reduced to 1MIU if the long-term therapy was required. Besides, a useful clinical application for the IRGPI was to screen potential compounds by the structure-based molecular docking methodology.

## Data availability statement

The datasets presented in this study can be found in online repositories. The names of the repositories and accession numbers can be found in the article/Supplementary material.

## Ethics statement

Ethical review and approval were not required for the study on human participants in accordance with the local legislation and institutional requirements. Written informed consent for participation was not required for this study in accordance with the national legislation and the institutional requirements.

## Author contributions

CW and RX designed the study. CW collected all the data. CW, YZ, and SL carried out the data processing and analyzing. CL, WC, and SJ led the statistical analysis. All authors interpreted data, wrote the manuscript, and approved the final version.

## Footnotes

1
https://www.ncbi.nlm.nih.gov/geo/

2
https://www.ebi.ac.uk/arrayexpress/

3
https://www.immport.org/

4
https://www.innatedb.com/

5
http://string-db.org

6
https://proteins.plus/

7
https://go.drugbank.com/

8
https://www.rcsb.org/

9
https://www.immport.org/shared/genelists

10
http://innatedb.com/download/innatedb_curated_genes.xls

## References

[B1] Agnello L. Colletti T. Lo Sasso B. Vidali M. Spataro R. Gambino C. M. (2021). Tau protein as a diagnostic and prognostic biomarker in amyotrophic lateral sclerosis. *Eur. J. Neurol.* 28 1868–1875. 10.1111/ene.14789 33638255

[B2] Agosta F. Altomare D. Festari C. Orini S. Gandolfo F. Boccardi M. (2018). Clinical utility of FDG-PET in amyotrophic lateral sclerosis and Huntington’s disease. *Eur. J. Nucl. Med. Mol. Imaging* 45 1546–1556. 10.1007/s00259-018-4033-0 29717332

[B3] Angelini D. F. De Angelis F. Vacca V. Piras E. Parisi C. Nutini M. (2020). Very early involvement of innate immunity in peripheral nerve degeneration in SOD1-G93A mice. *Front. Immunol.* 11:575792. 10.3389/fimmu.2020.575792 33329541 PMC7714949

[B4] Asano T. Meguri Y. Yoshioka T. Kishi Y. Iwamoto M. Nakamura M. (2017). PD-1 modulates regulatory T-cell homeostasis during low-dose interleukin-2 therapy. *Blood* 129 2186–2197. 10.1182/blood-2016-09-741629 28151427 PMC5391624

[B5] Beers D. R. Zhao W. Liao B. Kano O. Wang J. Huang A. (2011). Neuroinflammation modulates distinct regional and temporal clinical responses in ALS mice. *Brain Behav. Immun.* 25 1025–1035. 10.1016/j.bbi.2010.12.008 21176785 PMC3096756

[B6] Berman H. M. Westbrook J. Feng Z. Gilliland G. Bhat T. N. Weissig H. (2000). The protein data bank. *Nucleic Acids Res.* 28 235–242. 10.1093/nar/28.1.235 10592235 PMC102472

[B7] Bhattacharya S. Dunn P. Thomas C. G. Smith B. Schaefer H. Chen J. (2018). ImmPort, toward repurposing of open access immunological assay data for translational and clinical research. *Sci. Data* 5:180015. 10.1038/sdata.2018.15 29485622 PMC5827693

[B8] Breuer K. Foroushani A. K. Laird M. R. Chen C. Sribnaia A. Lo R. (2013). InnateDB: systems biology of innate immunity and beyond–recent updates and continuing curation. *Nucleic Acids Res.* 41 D1228–D1233. 10.1093/nar/gks1147 23180781 PMC3531080

[B9] Busse D. de la Rosa M. Hobiger K. Thurley K. Flossdorf M. Scheffold A. (2010). Competing feedback loops shape IL-2 signaling between helper and regulatory T lymphocytes in cellular microenvironments. *Proc. Natl. Acad. Sci. U.S.A.* 107 3058–3063. 10.1073/pnas.0812851107 20133667 PMC2840293

[B10] Calvo A. C. Cibreiro G. A. Merino P. T. Roy J. F. Galiana A. Rufián A. J. (2019). Collagen XIX Alpha 1 Improves Prognosis in Amyotrophic Lateral Sclerosis. *Aging Dis.* 10 278–292. 10.14336/AD.2018.0917 31011479 PMC6457048

[B11] Camu W. Mickunas M. Veyrune J.-L. Payan C. Garlanda C. Locati M. (2020). Repeated 5-day cycles of low dose aldesleukin in amyotrophic lateral sclerosis (IMODALS): A phase 2a randomised, double-blind, placebo-controlled trial. *EBioMedicine* 59:102844. 10.1016/j.ebiom.2020.102844 32651161 PMC7502670

[B12] Chiot A. Zaïdi S. Iltis C. Ribon M. Berriat F. Schiaffino L. (2020). Modifying macrophages at the periphery has the capacity to change microglial reactivity and to extend ALS survival. *Nat. Neurosci.* 23 1339–1351. 10.1038/s41593-020-00718-z 33077946

[B13] Chitnis T. Weiner H. L. (2017). CNS inflammation and neurodegeneration. *J. Clin. Invest.* 127 3577–3587. 10.1172/JCI90609 28872464 PMC5617655

[B14] Devigili G. Uçeyler N. Beck M. Reiners K. Stoll G. Toyka K. V. (2011). Vasculitis-like neuropathy in amyotrophic lateral sclerosis unresponsive to treatment. *Acta Neuropathol.* 122 343–352. 10.1007/s00401-011-0837-8 21626035

[B15] DiPALS Writing Committee and DiPALS Study Group Collaborators (2015). Safety and efficacy of diaphragm pacing in patients with respiratory insufficiency due to amyotrophic lateral sclerosis (DiPALS): a multicentre, open-label, randomised controlled trial. *Lancet Neurol.* 14 883–892. 10.1016/S1474-4422(15)00152-0 26234554

[B16] Feng T.-T. Zou T. Wang X. Zhao W.-F. Qin A.-L. (2017). Clinical significance of changes in the Th17/Treg ratio in autoimmune liver disease. *World J. Gastroenterol.* 23 3832–3838. 10.3748/wjg.v23.i21.3832 28638223 PMC5467069

[B17] Figueroa-Romero C. Monteagudo A. Murdock B. J. Famie J. P. Webber-Davis I. F. Piecuch C. E. (2022). Tofacitinib suppresses natural killer cells in vitro and in vivo: implications for amyotrophic lateral sclerosis. *Front. Immunol.* 13:773288. 10.3389/fimmu.2022.773288 35197969 PMC8859451

[B18] Gaur N. Huss E. Prell T. Steinbach R. Guerra J. Srivastava A. (2021). Monocyte-derived macrophages contribute to Chitinase Dysregulation in amyotrophic lateral sclerosis: a pilot study. *Front. Neurol.* 12:629332. 10.3389/fneur.2021.629332 34054686 PMC8160083

[B19] Gijselinck I. Van Mossevelde S. van der Zee J. Sieben A. Engelborghs S. De Bleecker J. (2016). The C9orf72 repeat size correlates with onset age of disease. DNA methylation and transcriptional downregulation of the promoter. *Mol. Psychiatry* 21 1112–1124. 10.1038/mp.2015.159 26481318 PMC4960451

[B20] Gilgun-Sherki Y. Panet H. Melamed E. Offen D. (2003). Riluzole suppresses experimental autoimmune encephalomyelitis: implications for the treatment of multiple sclerosis. *Brain Res.* 989 196–204. 10.1016/s0006-8993(03)03343-2 14556941

[B21] Giovannelli I. Bayatti N. Brown A. Wang D. Mickunas M. Camu W. (2021). Amyotrophic lateral sclerosis transcriptomics reveals immunological effects of low-dose interleukin-2. *Brain Commun.* 3:fcab141. 10.1093/braincomms/fcab141 34409288 PMC8364666

[B22] Gouel F. Timmerman K. Gosset P. Raoul C. Dutheil M. Jonneaux A. (2022). Whole and fractionated human platelet lysate biomaterials-based biotherapy induces strong neuroprotection in experimental models of amyotrophic lateral sclerosis. *Biomaterials* 280:121311. 10.1016/j.biomaterials.2021.121311 34952382

[B23] Groeneveld G. J. Van Kan H. J. M. Kalmijn S. Veldink J. H. Guchelaar H.-J. Wokke J. H. J. (2003). Riluzole serum concentrations in patients with ALS: associations with side effects and symptoms. *Neurology* 61 1141–1143. 10.1212/01.wnl.0000090459.76784.49 14581684

[B24] Holzer P. Farzi A. Hassan A. M. Zenz G. Jačan A. Reichmann F. (2017). Visceral inflammation and immune activation stress the brain. *Front. Immunol.* 8:1613. 10.3389/fimmu.2017.01613 29213271 PMC5702648

[B25] Hotta-Iwamura C. Benck C. Coley W. D. Liu Y. Zhao Y. Quiel J. A. (2018). Low CD25 on autoreactive Tregs impairs tolerance via low dose IL-2 and antigen delivery. *J. Autoimmun.* 90 39–48. 10.1016/j.jaut.2018.01.005 29439835 PMC5949247

[B26] Hoyer L. L. (2001). The ALS gene family of *Candida albicans*. *Trends Microbiol.* 9 176–180. 10.1016/s0966-842x(01)01984-9 11286882

[B27] Iguchi Y. Katsuno M. Niwa J.-I. Yamada S.-I. Sone J. Waza M. (2009). TDP-43 depletion induces neuronal cell damage through dysregulation of Rho family GTPases. *J. Biol. Chem.* 284 22059–22066. 10.1074/jbc.M109.012195 19535326 PMC2755930

[B28] Jakhar R. Sharma C. Paul S. Kang S. C. (2018). Immunosuppressive potential of astemizole against LPS activated T cell proliferation and cytokine secretion in RAW macrophages, zebrafish larvae and mouse splenocytes by modulating MAPK signaling pathway. *Int. Immunopharmacol.* 65 268–278. 10.1016/j.intimp.2018.10.014 30359933

[B29] Jiang L. Jiang H. Dai S. Chen Y. Song Y. Tang C. S.-M. (2022). Deviation from baseline mutation burden provides powerful and robust rare-variants association test for complex diseases. *Nucleic Acids Res.* 50:e34. 10.1093/nar/gkab1234 34931221 PMC8989543

[B30] Jin M. Günther R. Akgün K. Hermann A. Ziemssen T. (2020). Peripheral proinflammatory Th1/Th17 immune cell shift is linked to disease severity in amyotrophic lateral sclerosis. *Sci. Rep.* 10:5941. 10.1038/s41598-020-62756-8 32246039 PMC7125229

[B31] Kalim K. W. Yang J.-Q. Li Y. Meng Y. Zheng Y. Guo F. (2018). Reciprocal regulation of glycolysis-driven Th17 pathogenicity and regulatory T cell stability by Cdc42. *J. Immunol.* 200 2313–2326. 10.4049/jimmunol.1601765 29440353 PMC5860966

[B32] Kirby J. Ning K. Ferraiuolo L. Heath P. R. Ismail A. Kuo S.-W. (2011). Phosphatase and tensin homologue/protein kinase B pathway linked to motor neuron survival in human superoxide dismutase 1-related amyotrophic lateral sclerosis. *Brain* 134 506–517. 10.1093/brain/awq345 21228060 PMC3030763

[B33] Klemann C. J. H. M. Visser J. E. Van Den Bosch L. Martens G. J. M. Poelmans G. (2018). Integrated molecular landscape of amyotrophic lateral sclerosis provides insights into disease etiology. *Brain Pathol.* 28 203–211. 10.1111/bpa.12485 28035716 PMC8028446

[B34] Koch J. C. Tatenhorst L. Roser A.-E. Saal K.-A. Tönges L. Lingor P. (2018). ROCK inhibition in models of neurodegeneration and its potential for clinical translation. *Pharmacol Ther.* 189 1–21. 10.1016/j.pharmthera.2018.03.008 29621594

[B35] Komine O. Yamashita H. Fujimori-Tonou N. Koike M. Jin S. Moriwaki Y. (2018). Innate immune adaptor TRIF deficiency accelerates disease progression of ALS mice with accumulation of aberrantly activated astrocytes. *Cell Death Differ.* 25 2130–2146. 10.1038/s41418-018-0098-3 29568058 PMC6261996

[B36] Kučić N. Rački V. Šverko R. Vidović T. Grahovac I. Mršić-Pelčić J. (2021). Immunometabolic modulatory role of Naltrexone in BV-2 microglia cells. *Int. J. Mol. Sci.* 22:8429. 10.3390/ijms22168429 34445130 PMC8395119

[B37] Levin A. M. Bates D. L. Ring A. M. Krieg C. Lin J. T. Su L. (2012). Exploiting a natural conformational switch to engineer an interleukin-2 “superkine.” *Nature* 484 529–533. 10.1038/nature10975 22446627 PMC3338870

[B38] Li C. Yang W. Wei Q. Shang H. (2020). Causal association of leukocytes count and amyotrophic lateral sclerosis: a Mendelian randomization study. *Mol. Neurobiol.* 57 4622–4627. 10.1007/s12035-020-02053-7 32770314

[B39] Li J. J. Wang B. Kodali M. C. Chen C. Kim E. Patters B. J. (2018). In vivo evidence for the contribution of peripheral circulating inflammatory exosomes to neuroinflammation. *J. Neuroinflam.* 15:8. 10.1186/s12974-017-1038-8 29310666 PMC5759808

[B40] Lincecum J. M. Vieira F. G. Wang M. Z. Thompson K. De Zutter G. S. Kidd J. (2010). From transcriptome analysis to therapeutic anti-CD40L treatment in the SOD1 model of amyotrophic lateral sclerosis. *Nat. Genet.* 42 392–399. 10.1038/ng.557 20348957

[B41] Liu B.-S. Ferreira R. Lively S. Schlichter L. C. (2013). Microglial SK3 and SK4 currents and activation state are modulated by the neuroprotective drug, riluzole. *J. Neuroimmune Pharmacol.* 8 227–237. 10.1007/s11481-012-9365-0 22527636

[B42] Liu T. Zhang L. Joo D. Sun S.-C. (2017). NF-κB signaling in inflammation. *Signal Transduct. Target Ther.* 2:17023. 10.1038/sigtrans.2017.23 29158945 PMC5661633

[B43] Liu Y. Xie X. Xia L.-P. Lv H. Lou F. Ren Y. (2017). Peripheral immune tolerance alleviates the intracranial lipopolysaccharide injection-induced neuroinflammation and protects the dopaminergic neurons from neuroinflammation-related neurotoxicity. *J. Neuroinflam.* 14:223. 10.1186/s12974-017-0994-3 29145874 PMC5693474

[B44] Magen I. Yacovzada N. S. Yanowski E. Coenen-Stass A. Grosskreutz J. Lu C.-H. (2021). Circulating miR-181 is a prognostic biomarker for amyotrophic lateral sclerosis. *Nat. Neurosci.* 24 1534–1541. 10.1038/s41593-021-00936-z 34711961

[B45] Mao W. Lou Y.-F. Ye B. Lin S. Chen Y. Chen Y. (2012). Changes in peripheral CD4+CD25(high) regulatory T cells in the acute-on-chronic liver failure patients with plasma exchange treatment. *Inflammation* 35 436–444. 10.1007/s10753-011-9333-5 21505810

[B46] McCombe P. A. Lee J. D. Woodruff T. M. Henderson R. D. (2020). The peripheral immune system and amyotrophic lateral sclerosis. *Front. Neurol.* 11:279. 10.3389/fneur.2020.00279 32373052 PMC7186478

[B47] Munsat T. L. Andres P. L. Finison L. Conlon T. Thibodeau L. (1988). The natural history of motoneuron loss in amyotrophic lateral sclerosis. *Neurology* 38 409–413. 10.1212/wnl.38.3.409 3347345

[B48] Murdock B. J. Bender D. E. Kashlan S. R. Figueroa-Romero C. Backus C. Callaghan B. C. (2016). Increased ratio of circulating neutrophils to monocytes in amyotrophic lateral sclerosis. *Neurol. Neuroimmunol. Neuroinflamm.* 3:e242. 10.1212/NXI.0000000000000242 27308304 PMC4897983

[B49] Murdock B. J. Famie J. P. Piecuch C. E. Raue K. D. Mendelson F. E. Pieroni C. H. (2021). NK cells associate with ALS in a sex- and age-dependent manner. *JCI Insight* 6:147129. 10.1172/jci.insight.147129 33974561 PMC8262328

[B50] Murdock B. J. Zhou T. Kashlan S. R. Little R. J. Goutman S. A. Feldman E. L. (2017). Correlation of peripheral immunity with rapid amyotrophic lateral sclerosis progression. *JAMA Neurol.* 74 1446–1454. 10.1001/jamaneurol.2017.2255 28973548 PMC5822195

[B51] Newman A. M. Steen C. B. Liu C. L. Gentles A. J. Chaudhuri A. A. Scherer F. (2019). Determining cell type abundance and expression from bulk tissues with digital cytometry. *Nat. Biotechnol.* 37 773–782. 10.1038/s41587-019-0114-2 31061481 PMC6610714

[B52] Ravits J. M. La Spada A. R. (2009). ALS motor phenotype heterogeneity, focality, and spread: deconstructing motor neuron degeneration. *Neurology* 73 805–811. 10.1212/WNL.0b013e3181b6bbbd 19738176 PMC2739608

[B53] Rotolo R. A. Demuro J. Drummond G. Little C. Johns L. D. Betz A. J. (2021). Prophylactic exposure to oral riluzole reduces the clinical severity and immune-related biomarkers of experimental autoimmune encephalomyelitis. *J. Neuroimmunol.* 356:577603. 10.1016/j.jneuroim.2021.577603 33992861

[B54] Shah W. Yan X. Jing L. Zhou Y. Chen H. Wang Y. (2011). A reversed CD4/CD8 ratio of tumor-infiltrating lymphocytes and a high percentage of CD4(+)FOXP3(+) regulatory T cells are significantly associated with clinical outcome in squamous cell carcinoma of the cervix. *Cell Mol. Immunol.* 8 59–66. 10.1038/cmi.2010.56 21200385 PMC4002991

[B55] Shannon P. Markiel A. Ozier O. Baliga N. S. Wang J. T. Ramage D. (2003). Cytoscape: a software environment for integrated models of biomolecular interaction networks. *Genome Res.* 13 2498–2504. 10.1101/gr.1239303 14597658 PMC403769

[B56] Shirani A. Okuda D. T. Stüve O. (2016). Therapeutic advances and future prospects in progressive forms of multiple sclerosis. *Neurotherapeutics* 13 58–69. 10.1007/s13311-015-0409-z 26729332 PMC4720678

[B57] Sidaway P. (2017). Motor neuron disease: Peripheral immune cell levels correlate with disease progression in ALS. *Nat. Rev. Neurol.* 13:708. 10.1038/nrneurol.2017.149 29027543

[B58] Song Z. Zhou Y. Han X. Qin J. Tang X. (2021). Recent advances in enzymeless-based electrochemical sensors to diagnose neurodegenerative diseases. *J. Mater Chem. B* 9 1175–1188. 10.1039/d0tb02745f 33458727

[B59] Sun J. Huang T. Debelius J. W. Fang F. (2021). Gut microbiome and amyotrophic lateral sclerosis: A systematic review of current evidence. *J. Intern. Med.* 290 758–788. 10.1111/joim.13336 34080741

[B60] Swindell W. R. Kruse C. P. S. List E. O. Berryman D. E. Kopchick J. J. (2019). ALS blood expression profiling identifies new biomarkers, patient subgroups, and evidence for neutrophilia and hypoxia. *J. Transl. Med.* 17:170. 10.1186/s12967-019-1909-0 31118040 PMC6530130

[B61] Szklarczyk D. Morris J. H. Cook H. Kuhn M. Wyder S. Simonovic M. (2017). The STRING database in 2017: quality-controlled protein-protein association networks, made broadly accessible. *Nucleic Acids Res.* 45 D362–D368. 10.1093/nar/gkw937 27924014 PMC5210637

[B62] Tao H. Mimura Y. Aoe K. Kobayashi S. Yamamoto H. Matsuda E. (2012). Prognostic potential of FOXP3 expression in non-small cell lung cancer cells combined with tumor-infiltrating regulatory T cells. *Lung Cancer* 75 95–101. 10.1016/j.lungcan.2011.06.002 21719142

[B63] Thompson A. G. Gray E. Thézénas M.-L. Charles P. D. Evetts S. Hu M. T. (2018). Cerebrospinal fluid macrophage biomarkers in amyotrophic lateral sclerosis. *Ann. Neurol.* 83 258–268. 10.1002/ana.25143 29331073

[B64] Toljan K. Vrooman B. (2018). Low-Dose Naltrexone (LDN)-review of therapeutic utilization. *Med. Sci.* 6:E82. 10.3390/medsci6040082 30248938 PMC6313374

[B65] Tönges L. Günther R. Suhr M. Jansen J. Balck A. Saal K.-A. (2014). Rho kinase inhibition modulates microglia activation and improves survival in a model of amyotrophic lateral sclerosis. *Glia* 62 217–232. 10.1002/glia.22601 24311453

[B66] Trott O. Olson A. J. (2010). AutoDock Vina: improving the speed and accuracy of docking with a new scoring function, efficient optimization, and multithreading. *J. Comput. Chem.* 31 455–461. 10.1002/jcc.21334 19499576 PMC3041641

[B67] Vahsen B. F. Gray E. Thompson A. G. Ansorge O. Anthony D. C. Cowley S. A. (2021). Non-neuronal cells in amyotrophic lateral sclerosis - from pathogenesis to biomarkers. *Nat. Rev. Neurol.* 17 333–348. 10.1038/s41582-021-00487-8 33927394

[B68] van Rheenen W. Diekstra F. P. Harschnitz O. Westeneng H.-J. van Eijk K. R. Saris C. G. J. (2018). Whole blood transcriptome analysis in amyotrophic lateral sclerosis: A biomarker study. *PLoS One* 13:e0198874. 10.1371/journal.pone.0198874 29939990 PMC6016933

[B69] Wishart D. S. Feunang Y. D. Guo A. C. Lo E. J. Marcu A. Grant J. R. (2018). DrugBank 5.0: a major update to the DrugBank database for 2018. *Nucleic Acids Res.* 46 D1074–D1082. 10.1093/nar/gkx1037 29126136 PMC5753335

[B70] Yamada S. Hashizume A. Hijikata Y. Ito D. Kishimoto Y. Iida M. (2021). Ratio of urinary N-terminal titin fragment to urinary creatinine is a novel biomarker for amyotrophic lateral sclerosis. *J. Neurol. Neurosurg. Psychiatry* 92 1072–1079. 10.1136/jnnp-2020-324615 33737450

[B71] Yan J. Xu L. Welsh A. M. Chen D. Hazel T. Johe K. (2006). Combined immunosuppressive agents or CD4 antibodies prolong survival of human neural stem cell grafts and improve disease outcomes in amyotrophic lateral sclerosis transgenic mice. *Stem Cells* 24 1976–1985. 10.1634/stemcells.2005-0518 16644922

[B72] Yu D. Cook M. C. Shin D.-M. Silva D. G. Marshall J. Toellner K.-M. (2008). Axon growth and guidance genes identify T-dependent germinal centre B cells. *Immunol. Cell Biol.* 86 3–14. 10.1038/sj.icb.7100123 17938642

[B73] Zhang J. Huang P. Wu C. Liang H. Li Y. Zhu L. (2018). Preliminary observation about alteration of proteins and their potential functions in spinal cord of SOD1 G93A transgenic mice. *Int. J. Biol. Sci.* 14 1306–1320. 10.7150/ijbs.26829 30123078 PMC6097476

[B74] Zhao W. Beers D. R. Thonhoff J. R. Thome A. D. Faridar A. Wang J. (2020). Immunosuppressive functions of M2 macrophages derived from iPSCs of patients with ALS and healthy controls. *iScience* 23:101192. 10.1016/j.isci.2020.101192 32521508 PMC7286967

[B75] Zhou Q. Mareljic N. Michaelsen M. Parhizkar S. Heindl S. Nuscher B. (2020). Active poly-GA vaccination prevents microglia activation and motor deficits in a C9orf72 mouse model. *EMBO Mol. Med.* 12:e10919. 10.15252/emmm.201910919 31858749 PMC7005532

[B76] Zhou Y.-J. Wang H. Sui H.-H. Li L. Zhou C.-L. Huang J.-J. (2016). Inhibitory effect of baicalin on allergic response in ovalbumin-induced allergic rhinitis guinea pigs and lipopolysaccharide-stimulated human mast cells. *Inflamm. Res.* 65 603–612. 10.1007/s00011-016-0943-0 27043920

