# Supplementary figures and images for: Identification of an immune-related gene prognostic index for predicting prognosis, immunotherapeutic efficacy, and candidate drugs in amyotrophic lateral sclerosis
Source: Front Cell Neurosci. 2022 Dec 15;16:993424. doi: 10.3389/fncel.2022.993424 (PMC9798295; doi:10.3389/fncel.2022.993424)

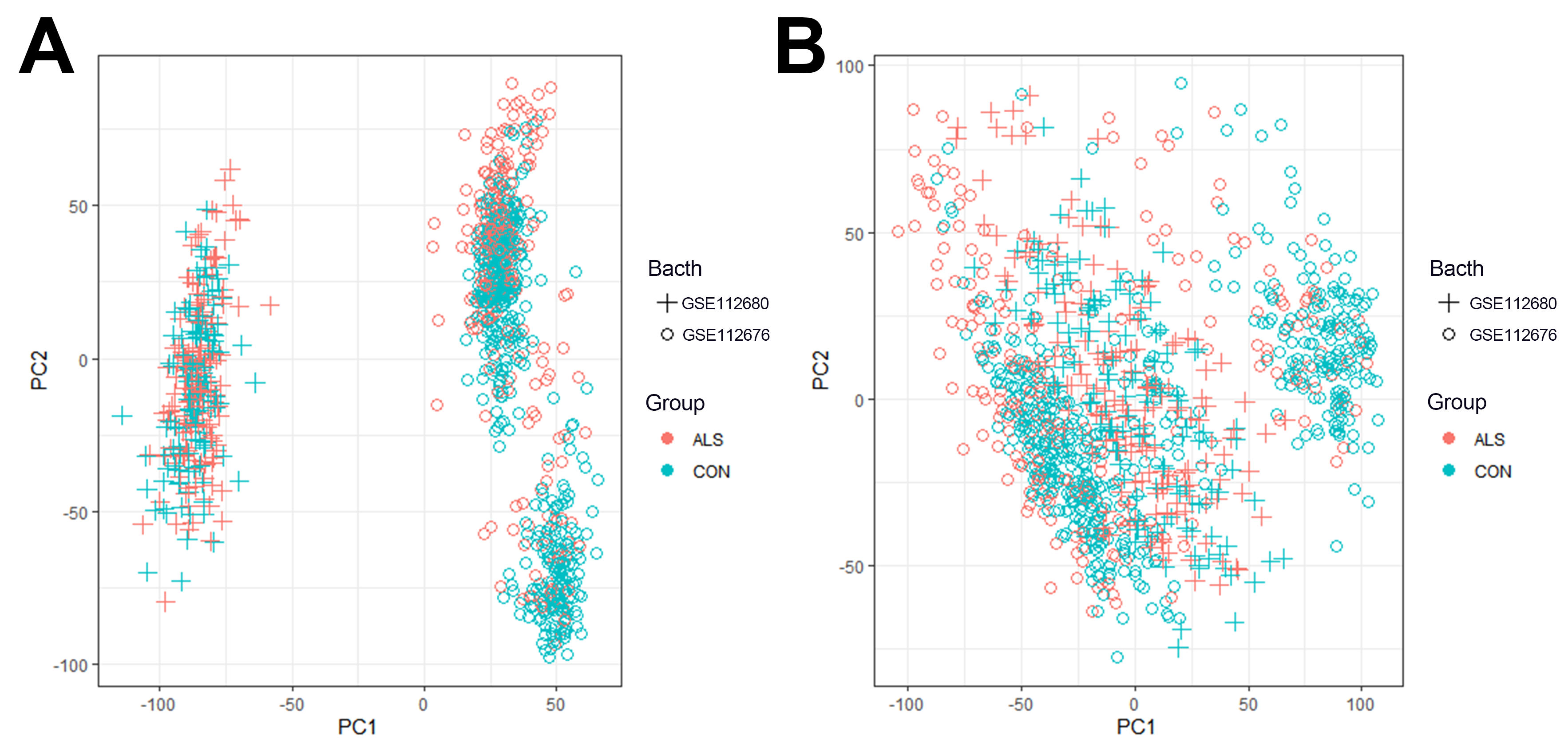

Supplement: Supplementary file 1 [file Image_1.TIF]

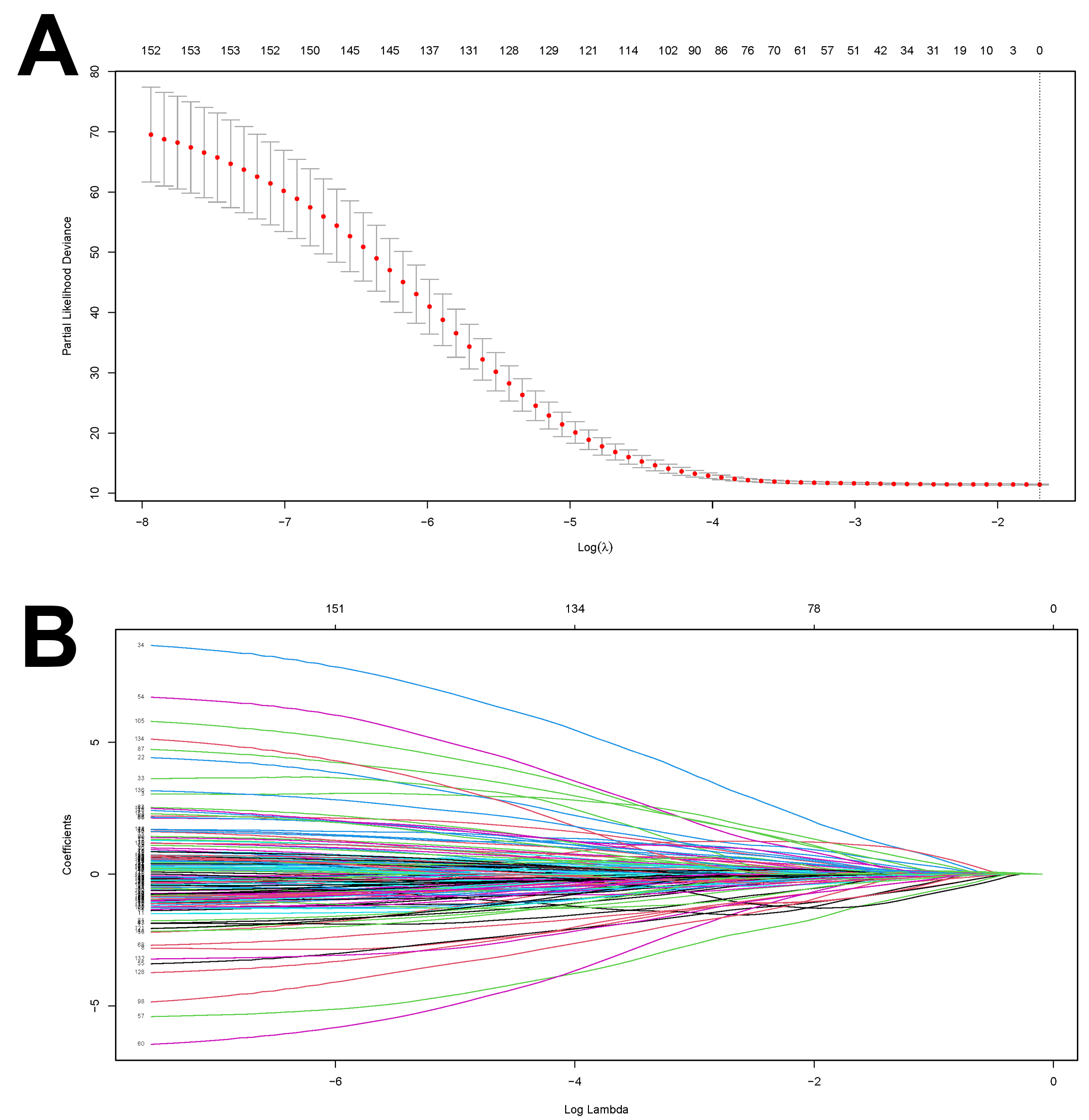

Supplement: Supplementary file 2 [file Image_2.TIF]

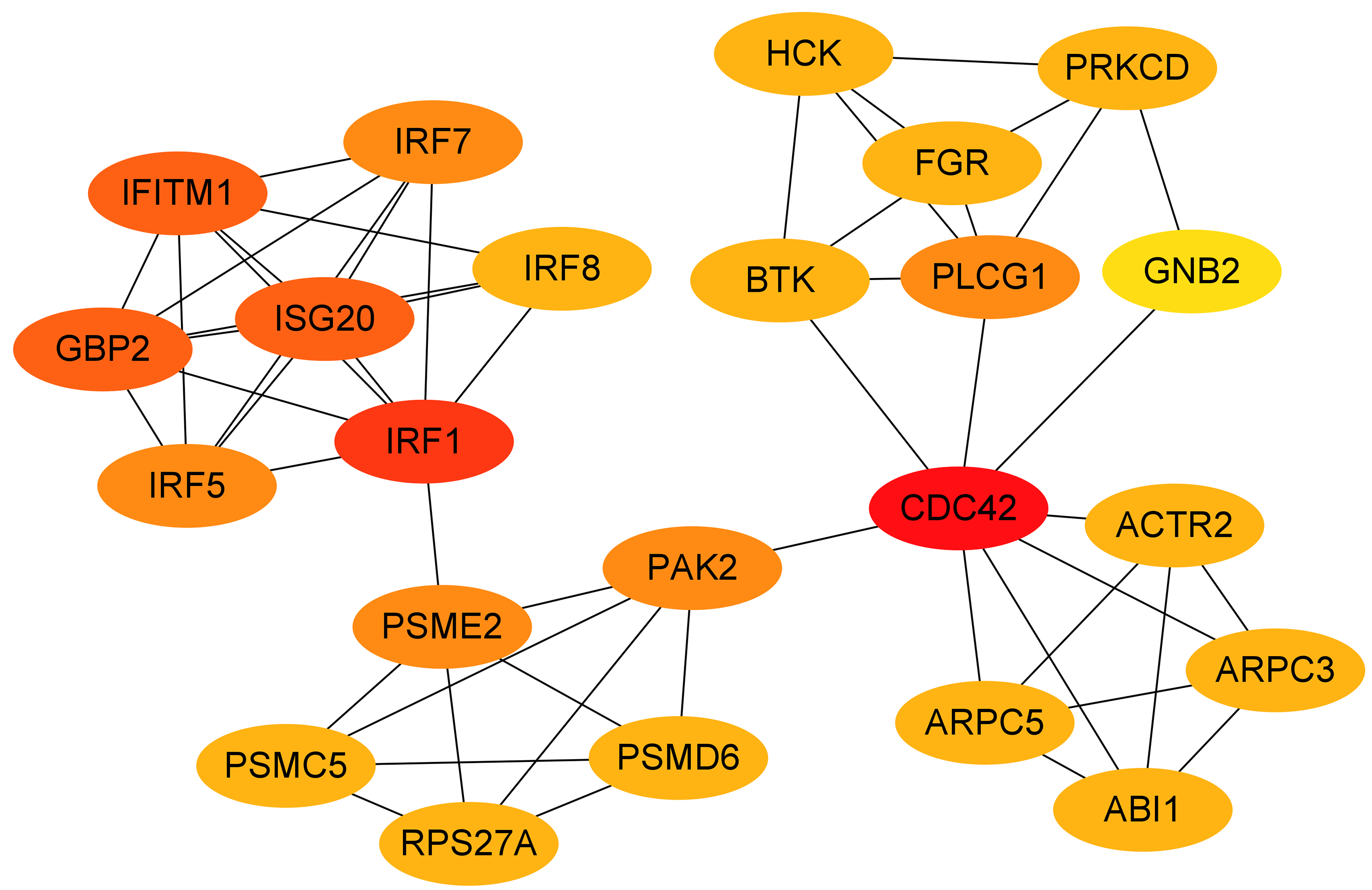

Supplement: Supplementary file 3 [file Image_3.TIF]
